# Multiple‐micronutrient supplementation: Evidence from large‐scale prenatal programmes on coverage, compliance and impact

**DOI:** 10.1111/mcn.12531

**Published:** 2017-12-22

**Authors:** Cristiana Berti, Michelle F. Gaffey, Zulfiqar A. Bhutta, Irene Cetin

**Affiliations:** ^1^ Department of Biomedical and Clinical Sciences, School of Medicine University of Milan Milan Italy; ^2^ Centre for Global Child Health The Hospital for Sick Children Toronto Ontario Canada; ^3^ Dalla Lana School of Public Health University of Toronto Toronto Ontario Canada; ^4^ Department of Nutritional Sciences and Department of Paediatrics University of Toronto Toronto Ontario Canada; ^5^ Center of Excellence in Women and Child Health Aga Khan University Karachi Pakistan

**Keywords:** communication, community, large‐scale implementation, micronutrients, pregnancy, WHO/CDC logic‐model

## Abstract

Micronutrient deficiencies during pregnancy pose important challenges for public‐health, given the potential adverse outcomes not only during pregnancy but across the life‐course. Provision of iron‐folic acid (IFA) supplements is the strategy most commonly practiced and recommended globally. How to successfully implement IFA and multiple micronutrient supplementation interventions among pregnant women and to achieve sustainable/permanent solutions to prenatal micronutrient deficiencies remain unresolved issues in many countries. This paper aims to analyse available experiences of prenatal IFA and multiple micronutrient interventions to distil learning for their effective planning and large‐scale implementation. Relevant articles and programme‐documentation were comprehensively identified from electronic databases, websites of major‐agencies and through hand‐searching of relevant documents. Retrieved documents were screened and potentially relevant reports were critically examined by the authors with the aim of identifying a set of case studies reflecting regional variation, a mix of implementation successes and failures, and a mix of programmes and large‐scale experimental studies. Information on implementation, coverage, compliance, and impact was extracted from reports of large‐scale interventions in Central America, Southeast Asia, South Asia, and Sub‐Saharan Africa. The *WHO/CDC Logic‐Model for Micronutrient Interventions in Public Health* was used as an organizing framework for analysing and presenting the evidence. Our findings suggest that to successfully implement supplementation interventions and achieve sustainable‐permanent solutions efforts must focus on factors and processes related to quality, cost‐effectiveness, coverage, utilization, demand, outcomes, impacts, and sustainability of programmes including strategic analysis, management, collaborations to pilot a project, and careful monitoring, midcourse corrections, supervision and logistical‐support to gradually scaling it up.

## 1 INTRODUCTION

Micronutrient deficiencies during pregnancy pose important challenges for public health, given the potential adverse outcomes not only during pregnancy but also across the life‐course (Bhutta, Salam & Das, 2013). The strategy universally most recommended and practised in pregnancy is the daily provision of iron‐folic acid (IFA) supplements delivered through ante‐natal care, with the priority focus being to combat maternal anaemia and iron deficiency, and to reduce the risk of low birth‐weight (WHO, 2012). Efforts are also increasing to optimize weekly IFA supplementation targeting nonpregnant women and adolescents (Aguayo, Paintal, & Singh, 2013; Vir, Singh, Nigam, & Jain, 2008), to be used as a preventive rather than a therapeutic measure for improving iron status before pregnancy and preventing anaemia in pregnancy. Preconception care has the potential to positively impact a million pregnancies worldwide each year (Berti et al., 2017; Dean et al., 2013). In many countries, however, little progress has been achieved in ensuring adequate IFA coverage to improve pregnancy outcomes (Sanghvi, Harvey, & Wainwright, 2010). The increasing concern that IFA alone may not be sufficient to replenish the concurrent micronutrient deficiencies that often occur in pregnant women has encouraged the launch of multiple micronutrient (MMN) supplementation (Bhutta, Imdad, Ramakrishnan, & Martorell, 2012). Scientific evidence demonstrates that compared with IFA, MNN supplements during pregnancy exert similar beneficial effects on maternal anaemia (Bhutta et al., 2013), mean birth‐weight, incidence of both low birth‐weight and small for gestational age (Fall et al., 2009; West et al., 2014), and neural tube defects and congenital heart defects (Czeizel, 2011). Because distribution systems are already in place to deliver IFA tablets to pregnant women, MMN supplementation may be a relatively cost‐effective way of improving pregnancy outcomes in undernourished populations (Bhutta et al., 2012). Programmatic experiences of IFA supplementation may provide useful learning for the introduction of MMN supplementation. It is widely acknowledged that provision of micronutrient supplements is most effective when incorporated into a comprehensive context‐based approach that integrates multiple interventions, including community‐based supplement distribution, nutrition education, female/community empowerment, basic primary health care, sanitation, marketing activities, and national‐level advocacy (Berti, Faber, & Smuts, 2014). Yet, little is known about how best to deliver these key interventions at scale, to ensure that impacts are achieved (Menon et al., 2014). Indeed, it is crucial to create actionable knowledge of relevant issues, including conducting operational research to identify methods and practices that effectively impact quality, coverage, equity, utilization, demand, outcomes, and sustainability of nutrition programmes (Menon et al., 2014). Given the incumbent call for addressing technical and evidence gaps to support policy makers and stakeholders with guidance on micronutrient supplementation interventions in pregnancy, in 2015 the World Health Organization (WHO), with the United Nations Children's Fund and the Micronutrient Initiative, launched a technical consultation “Multiple micronutrient supplements in pregnancy: implementation considerations for successful integration into existing programmes”. The intention was to identify programmatic experiences of worth that were both successful and not, thereby extracting best practices for implementation and improved estimated adherence and sustainability. Purposely, in this paper evidence from large‐scale prenatal IFA and MMN supplementation interventions is reviewed and synthesised to highlight factors, determinants and, mostly, actions (i.e., methods and approaches) that contribute to supplementation programme success or failure, with the objective of distilling important learning for effective planning and large‐scale implementation in public health practice.

**Key messages**

- Micronutrient malnutrition persists among childbearing‐aged and pregnant women worldwide
- Actionable knowledge of relevant issues at various scales is a key factor to align efforts, optimize cost‐effectiveness and accelerate progress towards improved nutrition outcomes
- Strengthening or establishing national technical and intersectoral committees/partnership, and securing assistance from bilateral and international agencies are vital in programme scaling‐up and sustainability
- Systematic and professional training of health‐workers along with community motivation and mobilization are crucial for achieving coverage and compliance
- Creating awareness and knowledge about optimal nutrition before and during pregnancy augments the potential to beneficially impact pregnancy outcomes

## 2 METHODS

### 2.1 Search‐strategy

**Figure 1:**
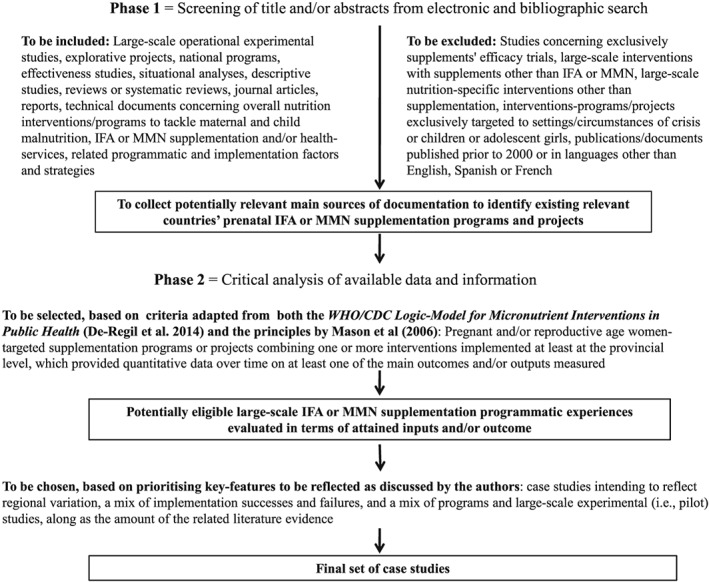
Outline of the methodological phases from screening titles/abstracts to selecting the final set of case studies. IFA = Iron‐Folic acid; MMN = Multiple Micronutrient

After prioritising the key‐topics to be reflected by the case studies, relevant articles and programme documentation were retrieved from a comprehensive search of PubMed/MEDLINE, Google‐indexed scientific literature, websites of major agencies, and through hand searching of the bibliographies of retrieved publications. Search terms included “effectiveness,” “nutrition,” “supplementation,” “micronutrient,” “programmes,” “pregnant women,” “implementation,” with no date or language limits. The strategy for selecting the final set of case studies for this review is schematised in Figure 1. After screening titles and/or abstracts for relevance, we retrieved the full‐text publications of large‐scale operational experimental studies and descriptive studies concerning nutrition interventions for reproductive‐age women and children, including IFA or MMN supplementation and/or other health services, and the related implementation factors and sustainability strategies, to identify existing relevant countries' programmes and projects (Phase 1). Only articles written in English, French or Spanish were retained. Next (Phase 2), we selected a subset of countries' experiences for this review by critically analysing available data and information based on the following criteria, derived from the *WHO/CDC Logic‐Model for Micronutrient Interventions in Public Health* (De‐Regil, Peña‐Rosas, Flores‐Ayala, & del Socorro Jefferds, 2014), and the principles proposed by Mason, Sanders, Musgrove, Soekirman, and Galloway (2006) for effective community health and nutrition programmes: programmes and large‐scale experimental studies (i.e., programmes combining one or more interventions implemented at least at the provincial or state level); targeting; available data on impact, that is, at least one of the main outcomes and/or outputs was measured over time (e.g., nutritional status, obstetric outcomes, coverage, adherence, awareness, etc.); and amount of information on example, implementation and challenges. Finally, to construct an objective picture based on a broad array of implementation features, we chose a final set of case studies intending to reflect regional variation, a mix of implementation successes and failures, and a mix of programmes and large‐scale experimental (i.e., pilot) studies, insofar as the availability of relevant information and data would allow.

### 2.2 Evidence‐synthesis

**Figure 2:**
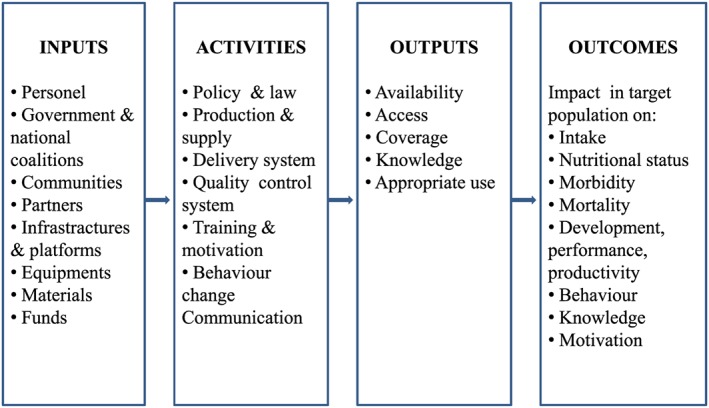
Conceptual framework of the *WHO/CDC Logic‐Model for Micronutrient Interventions in Public Health* highlighting indicators tied to expected intervention processes, as adapted from De‐Regil et al. (2014). The model is organised according to four main categories or components: Inputs: Resources invested in the intervention, including personnel, partnerships, politics and governance with different agendas, direct and indirect support from organizations, communities, and private sector, infrastructures, money, materials, and nutrition know‐out. Activities: Actions, events and processes of programme implementation such as developing protocols, passing legislation and regulation, designing production and supply delivery systems, engaging stakeholders, providing training, setting quality control systems, planning dissemination, education, counselling and advocacy communication strategies. Outputs: Direct effects or results of programme activities, such as procurement of annual supplies and availability of the supply in the country; staff trained and motivated to deliver and counsel participants on the intervention; availability of the intervention in communities or markets; and access and coverage to the intervention. Outcomes: Benefits or changes among target populations during or after the intervention in terms of the impact on both the micronutrient deficiency‐related issues (i.e., intake, nutritional status, morbidity and mortality, health functions), and the long‐term viability components (i.e., behaviours, knowledge, motivation, decision making, skills, individual/systemic/strategic/operational capacity etc.)

To organise findings and interpretations, we used a simplified version of the *WHO/CDC Logic‐Model for Micronutrient Interventions in Public Health*, adapted from the original (Figure 2). By explicitly outlining the different programme components (inputs, activities, outputs, outcomes) and how they related to each other to lead to the expected outcomes, the model served as an organizing framework to identify, analyse, and discuss the factors and methods contributing to success or failure in coverage, compliance, and impact within the selected case studies. After synthesising the available evidence pertaining to each component of the *WHO/CDC Logic‐Model*, we additionally considered other relevant factors important for prenatal micronutrient supplementation interventions that fall outside of the *WHO/CDC Logic‐Model*, including the importance of national nutrition surveys to initially inform micronutrient intervention design (Mason et al., 2006). Finally, where possible, we outlined brief conclusions and main lessons learned from the case studies about intervention implementation actions, including challenges as well as the evidence gaps. In particular, we sought to gather information that described the implementation methods and processes, including best practices in terms of content, supervision or monitoring.

## 3 RESULTS

Applying our inclusion and selection criteria to the potentially relevant records that we retrieved through our comprehensive search strategy, we ultimately selected a final set of eight case studies. Most retrieved documents were reviews and reports on nutrition‐sensitive programmes/interventions or nutrition‐specific interventions other than micronutrient supplementation, descriptive studies, action plans/roadmaps/guidelines exclusively describing implementation strategies and methods without any quantification of inputs and/or outcomes related to IFA or MMN supplementation, qualitative studies, and situational analyses with no data on programme/intervention effectiveness. In contrast, the most relevant sources of useful data and information were studies, reports, and summarising reviews of country cases' nutrition programmes, including micronutrient supplementation for women of reproductive age and children.

### 3.1 Overall shortcomings in the current state of evidence: What is missing

Our search revealed a paucity of literature documenting the impact of large‐scale integrated nutrition and health programmes on obstetric and foetal outcomes. Indeed, while many trials have considered the effects of IFA supplementation on pregnancy and birth outcomes (Lassi, Salam, Haider, & Bhutta, 2013; Peña‐Rosas, De‐Regil, Dowswell, & Viteri, 2012), our search retrieved no reports on these outcomes in the context of large‐scale IFA programmes. Additionally, we did not find any relevant paper that addressed exclusively challenging or failed supplementation programmes, although some cases are mentioned in literature. For example, South Africa was cited as a country where anaemia is still highly relevant even though IFA supplementation policies have been in place for some time (Deitchler, Mathys, Mason, Winichagoon, & Tuazon, 2004; Visser & Herselman, 2013), but neither quantitative data nor detailed analysis concerning barriers have been published. In other words, the available experiences tended to emphasize the programmes achievements, whereas little attention was given to programme faltering, barriers or shortcomings. Likewise, although there have been several large, population‐based trials of IFA and MMN supplementation in China in recent years (Liu et al., 2013; Wang, Pei, Song, Chen, & Zheng, 2013; Zeng et al., 2008), we could not find publications that focused in detail on the implementation aspects of these interventions. Moreover, because technical guidelines developed specifically for micronutrient deficiency are available as well as on‐going nutrition plans or roadmaps for Ethiopia, South Africa, and Kenya, reports of related impacts and analyses of implementation issues are unlikely to be available for some years yet. Furthermore, our findings likely pointed out a lack of MMN or IFA supplementation programmes in high‐income countries given that only studies of single‐micronutrient interventions such as folic acid were found (Branum, Bailey, & Singer, 2013; Nilsen et al., 2006).

### 3.2 Selected case studies

All were considered successful with respect to implementation and impact. Their general characteristics are summarised in Table 1. We included one case from Central America, one from South Asia, five from Southeast Asia, and one from Sub‐Saharan Africa. Seven were case studies of IFA supplementation interventions, and one included both IFA and MMN. Three case studies were national programmes targeting pregnant women, and five were pilot studies targeting women of reproductive age (both pregnant and nonpregnant women). All evaluated the intervention impact on incidence of anaemia and/or maternal or women's haemoglobin level, except for the Indonesia pilot study. Only two pilot studies investigated the impact on other health outcomes, that is, the association between neonatal birth‐weight and an integrated deworming and IFA‐supplementation intervention in Northwest Vietnam, and between the reduction in early infant mortality and the use of skilled birth attendants in Indonesia. Below, we present evidence from each case study on effective implementation strategies adopted, in the attempt to identify key intervention factors, that is which way worked and which failed.

**Table 1:** General characteristic of the selected case studies.

Regional variation	Central America	Nicaragua (Mora, 2007)
	South Asia	Nepal (Pokharel et al., 2011)
	South‐East Asia	Philippines (Nutrition Reviews, 2005); Indonesia (Shankar et al., 2009); and Vietnam (Casey et al., 2010; Ninh et al., 2003; Nutrition Reviews, 2005; Passerini et al., 2012)
	Sub‐Saharan Africa	Ghana (MacDonald et al., 2007)
Design and targeting	National programmes targeting pregnant women	Nicaragua (Mora, 2007); Nepal (Pokharel et al., 2011); and Vietnam^a^ (Ninh et al., 2003)
	Pilot‐studies targeting women of reproductive age	Philippines (Nutrition Reviews, 2005); Indonesia (Shankar et al., 2009); Vietnam (Casey et al., 2010; Nutrition Reviews, 2005; Passerini et al., 2012); and Ghana (MacDonald et al., 2007)
Type and mode of supplementation	IFA	Nicaragua (Mora, 2007); Nepal (Pokharel et al., 2011); Vietnam (Casey et al., 2010; Ninh et al., 2003; Nutrition Reviews, 2005; Passerini et al., 2012); and Philippines (Nutrition Reviews, 2005); (MacDonald et al., 2007)
	IFA plus MMN	Indonesia (Shankar et al., 2009)
	Comprehensive approach with at least another intervention for anaemia control	Nicaragua (Mora, 2007); Nepal (Pokharel et al., 2011); and Vietnam (Casey et al., 2010; Ninh et al., 2003; Passerini et al., 2012)
Data on health outcomes	Anaemia and/or iron status	Nicaragua (Mora, 2007); Nepal (Pokharel et al., 2011); Vietnam (Casey et al., 2010; Ninh et al., 2003; Nutrition Reviews, 2005; Passerini et al., 2012); Philippines (Nutrition Reviews, 2005); and Ghana (MacDonald et al., 2007)
	Neonatal outcomes	Vietnam (Passerini et al., 2012) and Indonesia (Shankar et al., 2009)
Data on performance outcomes	Facilitators' quality	Indonesia (Shankar et al., 2009)
Data on outputs	Coverage	Nicaragua (Mora, 2007); Nepal (Pokharel et al., 2011); Vietnam (Ninh et al., 2003); Indonesia (Shankar et al., 2009); Casey et al., 2010; Passerini et al., 2012); and Ghana (MacDonald et al., 2007)
	Adherence	Nicaragua (Mora, 2007); Philippines (Nutrition Reviews, 2005); and Vietnam (Casey et al., 2010; Passerini et al., 2012)
	Antenatal care	Nepal (Pokharel et al., 2011) and Indonesia (Shankar et al., 2009)
	BCC strategies	Nicaragua (Mora, 2007); Philippines (Nutrition Reviews, 2005); and Vietnam (Nutrition Reviews, 2005)
	Sales	Philippines (Nutrition Reviews, 2005) and Vietnam (Nutrition Reviews, 2005)

IFA = Iron‐Folic acid; MMN = Multiple Micronutrient; BCC = Behavioural Change Communication

a Targeting both pregnant women and nonpregnant women.

#### 3.2.1 National programmes

##### *Nicaragua* (Mora, 2007)

The available literature evaluating the Nicaragua national Integrated Anaemia Control Strategy (IACS) provided the most detailed information on implementation and impact of all the case studies included. The IACS was developed and gradually improved, as part of a National Micronutrient Plan, with IFA supplementation to pregnant women provided as a part of a comprehensive approach including other strategies for anaemia control such as flour fortification; behavioural change communication (BCC); target‐training to health service and non‐governmental organization (NGO) personnel and community health volunteers; and development of a system for programme monitor and evaluation.

The key programme characteristics are listed in Appendix A. The IACS implementation succeeded in improving IFA supplementation's coverage and adherence and resulted in a significant reduction (~68%) of anaemia rates among women of reproductive age. The analysis of inputs and activities highlighted the presence of most factors acknowledged as successful for achieving favourable outputs and outcomes, including baseline country analyses, political commitment, and partnerships. The specific facilitative actions, that is, the strategic ways that contributed to the successful IACS implementation were oriented to: establishing and disseminating clear IFA supplementation policies and target‐technical guidelines among all the health care personnel; involving community health volunteers in supplement delivery, follow‐up and counselling, coupled with increasing knowledge of health care providers and community health volunteers on anaemia and supplementation; and creation of a coordination task‐force including several stakeholders; conducting operational research. In contrast, some activities were not properly engaged due to practical challenges, thereby preventing any conclusion on their final role in the expected results.

##### *Nepal* (Pokharel, Maharjan, Mathema, & Harvey, 2011)

The Iron Intensification Project within the Nepal National Plan of Action for the Control of Anaemia is an example of strong partnership with international non‐governmental agencies and organizations supporting IFA supplementation plus deworming to pregnant women as part of the ante‐natal care services. Detailed documentation is available. The Iron Intensification Project was developed and implemented by integrating activities aimed at improving intervention access via distribution of supplement and counselling supported by female community health volunteers; strengthening complementary measures such as dietary promotion and fortification at national level; providing high‐quality training; establishing intensive monitoring and supervision (Nepal Government, 2014; Nepal Ministry of Health and Population et al., 2012).

Components and subcomponents contributing to and constraining the achievement of outputs and outcomes are listed in Appendix A. In 2011, anaemia prevalence was 1.5‐fold lower than at baseline, even if slightly higher than in 2006. Over the implementation period, a substantial scaling‐up was documented with the programme succeeding to cover 75 districts and to raise the number of pregnant women taking IFA (~70%) and attending ante‐natal care (~45%). The critical implementation‐delivering actions towards the successful enhancement of the overall IFA supply and adherence likely relied mostly on the following: maintaining a pro‐active involvement of motivated female community health volunteers; establishing a strong coordination in skill‐based training focused on supporting pregnant women in attending ante‐natal care clinics and taking IFA; ensuring adequate IFA and deworming medicine supply, appropriate supervision of health‐workers and community health volunteers; monitoring all activities; central coordination of messaging to address anaemia; and carrying‐out operational research and producing strong and consistent evidence of on‐going effective implementation to confirm a gradual scale‐up.

##### *Vietnam* (Ninh, Khan, Vinh, & Khoi, 2003)

The national Iron‐Deficiency Anaemia Control Programme in Vietnam is an example of national IFA‐programme with some bottlenecks. The programme included universal‐daily IFA supplementation of pregnant women, weekly supplementation of children 6–15 years of age, supplementation of nonpregnant women (by local organizations including Women's Union and Youth Union); Information, Education and Communication (IEC) to create awareness of the role of iron in foetal growth and perinatal mortality; operational research on food biscuit and fish‐sauce fortification with other micronutrients and deworming for nonpregnant women.

The programme features are outlined in Appendix A. Several implementation strengths were evident in the programme planning. The programme was delivered through the existing preventive health system, and it promoted community participation in the programme's management. IEC activities were implemented widely, focusing on iron‐rich foods, to increase knowledge and awareness of the role of iron. Although a reduction of anaemia (~39%) was achieved in pregnant women, concurrently a significant decrease in programme coverage occurred in terms of both targeted women and benefited districts, probably due to lack of supplement supplies and low utilization of community health volunteers. In contrast, the survey in 2000 found out a likely role of IEC strategies in decreasing anaemia, as a significant difference in anaemia prevalence was seen between people who were and were not exposed to IEC messages. The overall shortage of information about activities and outputs, including adherence and IEC, limits understanding of the extent of the intervention impact on anaemia.

#### 3.2.2 Pilot studies

These studies were chosen as examples of operational research investigating the effectiveness of specific delivery‐platforms such as community‐based nutrition programmes, health system platforms integrating nutrition interventions, and/or market‐based interventions at achieving either process or impact outcomes. Appendix A shows the main elements and implementation features of each case as well as the main outputs and outcomes. A summary of campaigns and relevant actions undertaken in each case is described below.

##### *Western‐Pacific countries: Philippines and Vietnam* (Nutrition Reviews, 2005)

These one‐year pilot‐effectiveness projects were conducted jointly by Department of Health and United Laboratories (UNILAB), the Philippines' largest private pharmaceutical company, with the support of the WHO Regional Office for the Western Pacific. “Social marketing” was applied as a framework to be applied for selling and dispensing preventive‐IFA to women of reproductive age by community workers, and motivating them to both adopt and maintain the behaviour for a long period of time. These studies followed national nutrition surveys on the prevalence of anaemia among pregnant women. A master protocol was developed and adapted in each study based on contextual elements and rapid situation assessments, in order to study impact and processes of implementing a weekly‐supplementation approach in countries with high anaemia rates but different socioeconomic systems and cultures. Pregnant women were given pregnancy‐targeted UNILAB tablets free of charge until the 3rd month after the delivery, then were expected to purchase the UNILAB supplements specifically target to nonpregnant women. In Philippines, pregnant women were delivered with a monthly dose either at prenatal check‐ups in local health units or at home visits by a responsible trained volunteer health‐worker or midwife, whereas nonpregnant women could buy their specifically targeted supplements at the drugstore or directly by the local health staff. In Vietnam, nonpregnant women could buy their specifically targeted UNILAB tablets from Women's Union collaborators. Four surveys were conducted to evaluate Knowledge, Attitudes and Practices (KAP) towards anaemia and IFA tablets, and to collect haematological parameters with haemoglobin, ferritin, and soluble transferrin receptor being assessed by national institutes.

A part from an overall enhancement of iron status, the most salient results of these pilot studies (Appendix A) were the substantial rise in sales of the weekly IFA tablets and the effectiveness of the approach in remarkably improving KAP among women of reproductive age. In the Philippines, adherence progressively improved over time, moving from ~6% at the 1st survey to over 95% by the 4th survey. In Vietnam, the Women's Union network was crucial to overcome the shortfalls in the existing national programme distribution system and expand opportunities for communication and education among women of reproductive age. Overall, the key strategies and methods successfully employed were designed around: providing intensive social marketing and promotion with IEC materials, and creative events; actively mobilizing community leaders and mostly women organizations; orientation or training of all the personnel involved in the project from national to provincial to rural level in social marketing and interpersonal communication skills; producing specifically‐targeted supplements for pregnant women and attractive supplements for women to consider “worth buying” by using country‐specific names evoking traditional concepts of woman's beauty; and selling supplements at affordable prices to nonpregnant women and secondary‐school girls through local drugstores and village health‐workers and women; regular monitoring and communication on project progress between project‐technical responsible and commune/village/organization leaders.

##### *Indonesia* (Shankar et al., 2009)

The Supplementation with Multiple Micronutrients Intervention Trial (SUMMIT) aimed to compare benefits of MMN versus IFA supplements on maternal and child health, and was designed as both a programme and research trial. Drawing on community‐based participatory methods in conjunction with iterative evidence‐based implementation enhancements, SUMMIT attempted to enrich outputs and improve overall impacts. Early formative research on existing health care delivery and health‐seeking patterns preceded the project design.

Project outcomes (Appendix A) included a rise in the rate of skilled birth attendance among multiparous women, and a significant reduction in early infant mortality linked to the increased use of skilled birth attendants. The quality of community facilitators' performance was directly associated with the improving impact of the MMN supplement on infant health. Early ante‐natal care visits increased significantly. The most salient strategies successfully employed were on the basis of the following: enhancements to programme implementation driven by evidence; creation of a team of skilled, motivated and well‐performing community facilitators through rigorous evidence‐based recruitment processes and accurate performance evaluation, and regular retraining and program certifications.

##### *North*w*est Vietnam* (Casey et al., 2010; Passerini et al., 2012) *and Ghana* (MacDonald, Mildon, Neequaye, Namarika, & Yiannakis, 2007)

These pilots evaluated the impact on anaemia of preventive‐IFA supplementation combined with other health interventions within women of reproductive age.

Following a baseline survey, in two‐districts of a northern Vietnamese province a pilot project was established to make preventive‐IFA supplementation and regular deworming treatment freely and universally available for all women of reproductive age. Results (Appendix A) showed that the prevalence of low birth‐weight was significantly reduced in infants born to mothers who had access to a prepregnancy programme of regular deworming and weekly IFA supplementation. The project was effective in significantly and sustainably in reducing the prevalence of anaemia and maintaining high compliance rates over 3 years. Actions resulted successful comprised the following: integration within existing health system; training of district staff, commune health‐station nurses and village health‐workers; and rigorous and timely monitoring through an external quality control system.

The MICronutrients And Health (MICAH) programme was a NGO‐led large‐scale, multicountry, 10‐year programme led by World Vision Canada with funding from the Canadian International Development Agency to reduce prevalence of micronutrient deficiencies in women and children. It was implemented in five African countries. In Ghana, anaemia strategy focused mainly on improving iron intake by supplementation whereas, in contrast with the other MICAH countries, minor emphasis was put on preventing malaria and hookworm due to the low malaria prevalence at baseline. Results (Appendix A) showed that weekly IFA supplementation led to a significant decrease in the prevalence of anaemia in women of reproductive age. The key intervention factors which resulted important for this attainment were the following: evidence‐based, regularly monitored, rigorously evaluated, and comprehensive approach that led to design an effective, context‐specific anaemia prevention and control programme; motivation of community health volunteers, that is, recognising their role in the programme progresses using some kind of incentives; support IFA distribution and provide appropriate health‐counselling messages regarding anaemia and supplementation; and integration within existing health system.

## 4 DISCUSSION

Micronutrient malnutrition persists among pregnant women in many countries not only because the causes of under‐nutrition may be not successfully addressed (Berti et al., 2014; Bhutta, Das, et al., 2013) but also because the methodologies applied to deliver interventions are often inadequate (Menon et al., 2014). From existing programmes, it is already known which are the key features and actions essential to initiate and sustain a micronutrient supplementation programme (Mason et al., 2006). Nevertheless, coverage and adherence rates often remain low. Hence, existing experiences can also be analysed to understand the ways in which inputs and factors, that is, capacities and strategies, were specifically and sustainably implemented. In this review, by using the *WHO/CDC Logic‐Model for Micronutrient Interventions in Public Health* framework to evaluated programmatic components and subcomponents (what), we identified and summarised the actions and methods (how) which contributed to success in coverage, compliance, and impact within the selected case studies.

### 4.1 Shortfalls

The most relevant gaps observed in our case studies were in the national programmes. They mostly concerned an overall lack of data about BCC outputs, intermediate indicators, dietary intake, coverage, and adherence to IFA supplementation among pregnant women. These findings were in line with those highlighted by Mason et al. (2006) which pointed out how globally, impact evaluation, which refers to the net effects of interventions on changing outcomes, seemed to be lacking. Insufficient resources for routine laboratory diagnosis, weakness in delivering, monitoring, coordinating, supervision, and reporting systems for supplements' supply, distribution, and adherence, lack of consensus on daily or intermittent IFA‐supplementation policies for pregnant women among health‐professionals, confusing communication messaging were the key constraints faced within the case studies we reviewed. As bottlenecks for a better understanding of the extent of each subcomponent's impact on changing outcomes, the paucity of information about trends in outputs, additional activities and micronutrient status could compromise the effective implementation of interventions. Misleading messages almost became a barrier to advocating for IFA consumption in Nepal (Pokharel et al., 2011) and the social marketing pilot project in Vietnam (Nutrition Reviews, 2005).

### 4.2 Successful factors and strengths

The case studies we selected succeeded in reducing anaemia rates among both pregnant women and nonpregnant women. The analysis of inputs and activities highlighted the presence of factors acknowledged as successful for achieving favourable outputs and outcomes. Indeed, the successful planning of the IFA interventions was foreseen by accurate baseline country analyses aimed at documenting the iron deficiency's magnitude, identifying all the potential risk factors, assessing the status quo (i.e., available inputs/resources and existing policies) and understanding which capacities needed to be developed. These actions have been highlighted as crucial to align efforts and actions, and to accelerate progress towards improved nutrition‐health outcomes (Deitchler et al., 2004; Olney, Rawat, & Ruel, 2012; Pelletier et al., 2012). All programmes and projects we selected, provided supplementation interventions through context‐specific integrated delivery methods, by using existing delivery platforms. Developing context‐specific action plans, along with operation research aimed at addressing key constraints and establishing the feasibility of delivery models, was also shown to be useful for the proper sequencing of actions for scaling up effectively and sustainably. These elements helped optimize cost‐effectiveness and maximize available human resources, as also demonstrated in a systematic review about integration of targeted health interventions into health systems (Atun, de Jongh, Secci, Ohiri, & Adeyi, 2010). Furthermore, all experiences we reviewed had both high political advocacy and good governance, which were shown in West Africa to be vital in financing, research, policy‐making, and programme implementation (Sodjinou et al., 2014). Likewise, our cases showed national technical and intersectoral committees/partnership, and assistance from bilateral and international agencies. Government/Private sector/Community coalitions were particularly crucial to promote and expand the preventive IFA supplementation programme in the Western Pacific countries' pilot studies (Nutrition Reviews, 2005). Cases in Nicaragua (Mora, 2007), Nepal (Pokharel et al., 2011), Northwest Vietnam (Casey et al., 2010; Passerini et al., 2012) and Ghana (MacDonald et al., 2007) outlined the role of local and international NGOs as both active forces in filling public‐health system at macro and microlevels, capacity building of human resource, and implementing health activities. They covered a broad range of tasks from providing regular, intensive technical support and training, funds, staff and expert consultants to conducting monitoring and adequacy evaluations or undertaking large‐scale health interventions as observed in the MICAH programme (Berti, Mildon, Siekmans, Main, & Macdonald, 2010). Most importantly, the selected cases evidenced that during the implementation period, anaemia and potentially related complications such as maternal or infant mortality and low birth‐weight, showed a significant decrease that occurred concurrently to the increase of women of reproductive age taking IFA and attending ante‐natal care. The critical implementation delivering‐activities to enhance coverage and adherence included the following: training and delivery within existing programmes (using what is already in place reduces costs); measurement and recording of supplement supply and adherence (using fieldworkers and health‐workers), monitoring and supervisory systems; fixed time to deliver to home (home visits); and providing education and motivation to women (explaining benefits, side‐effects, asking to note benefits; creating awareness/knowledge).

#### 4.2.1 Strategic implementation actions: How were key intervention factors implemented effectively?

##### IEC strategies

BCC strategies to promote behaviours that support adequate intake, ensure availability of supply, and help to empower communities to be more self‐reliant are considered relevant factors to be implemented. A systematic analysis of approaches to social and BCC for preventing and reducing anaemia suggested that supplementation could succeed by creating awareness and knowledge about the optimal behaviours to reach health and well‐being within both consumers and all stakeholders involved (Lamstein et al., 2014). In our set of cases, the IEC implementation actions successfully employed improved the overall KAP concerning anaemia implications and IFA importance during pregnancy, as well as how to enhance supplements' iron‐absorption through diet. They consisted the following: carrying out, on a regular basis, popular educational and creative events at clubs, religious places, and shops; disseminating field‐tested communication materials among schoolgirls and women of reproductive age, as well as flipcharts and up‐to‐date manuals among health‐workers and community health volunteers; and promoting media‐messages by the means of nationwide campaigns. Particularly, the use of clear and simplified key messages both in verbal and pictorial communication mostly favoured IFA adherence whilst tackling misleading information among both pregnant women and health stakeholders, thus counteracting unanticipated negative influences. Strong coordination, supervision and field‐testing of communication materials and channels underlie these processes. A detailed description of IEC processes undertaken and promotional materials developed, including a picture of a promotional handout for women, in a pilot‐demonstration project aimed at reducing the burden of anaemia, and hookworm within the Northwest Vietnam case chosen for this review, was provided by Phuc et al. (2009).

##### Community and health‐system involvement

Findings gathered from our selected set of cases reinforced the recognition of the role of community mobilization and motivation in achieving results. The pilot studies in Western Pacific countries (Nutrition Reviews, 2005) provided the most interesting result as they demonstrate that anaemia prevention could be accomplished through preventive‐IFA supplementation when behavioural changes occur. Combining community mobilization and social marketing helped address the challenge to put a process in place capable of motivating women to both adopt and maintain the behaviour for a long period of time, thereby fostering and sustaining the demand. The most outstanding result was the relevant purchase of the weekly IFA tablets among nonpregnant women that reflected the increased positive attitude of respondents towards taking regularly IFA. Because it was unlikely governments could continuously provide weekly‐IFAs to all women of reproductive age, communities and women themselves had to actively participate in the process, with consumer demand becoming a fundamental programme‐element. Apparently, developing personnel well‐skilled in “social marketing and interpersonal communication” making supplements easily accessible and affordable, and shifting the women's perceptions about iron‐containing supplementations as medicines with side‐effects by the means of attractive marketing were the strategic ways which contributed to success.

The selected case studies also showed that involving community health volunteers in supplement delivery, follow‐up and counselling impacted beneficially on supplementation effectiveness and anaemia. Maintaining a proactive involvement of motivated female community health volunteers had several concomitant advantages. They affected coverage and adherence by reaching pregnant women through home visits to provide IFA supplements, counselling, referrals about IFA supplements collection on a monthly basis, and safe storage, educational materials, and follow‐up. Indeed, community health volunteers were shown to be able to extend health systems and basic services directly to communities and households, thereby reducing morbidity in several resource‐constrained settings (Columbia University Technical Report, 2011; Perry, Zulliger, & Rogers, 2014). As an example, in the Vietnamese social marketing project we selected (Nutrition Reviews, 2005), Women's Union collaborators filled the gaps in the existing national programme's IFA distribution system, which had contributed to the relevant decrease in programme coverage observed from 1995 to 2000 (Ninh et al., 2003). In the experiences we selected, the methods which appeared to facilitate supplement adherence consisted of delivering reminder pamphlets to women on how and when taking weekly tablets (e.g., always on the same week day before bedtime eating foods rich in iron and vitamin C) or dosing‐schedule tips, and developing supplements' covers including educational information and listings of recommended locally available iron‐rich foods. Furthermore, community health volunteers were effective for improving overall KAP concerning IFA, and promoting overall healthy behaviour among both expectant mothers and eventually their families. Community agents were also crucial in increasing ante‐natal care attendance by promoting its early use, which in turn allowed women to benefit from available package of services such as deworming medicine and skilled birth assistants. Only a well‐implemented and locally adaptable community health volunteer workforce can deliver highly cost‐effective interventions that improve maternal and child health (Singh & Sachs, 2013). The need to systematically and professionally manage, support, motivate and train lay community members, appropriately to context, task load and expectations, to be a part of the health workforce emerged as a core component of primary health care systems in low resource settings (Columbia University Technical Report, 2011; Solon, 2006). As an example, the Indonesian SUMMIT programme (Shankar et al., 2009) attempted to achieve impacts by prioritizing and systematically enhancing implementation activities which focused on training community facilitators and informal or formal leaders to widely spread information on the value of pregnancy ante‐natal care and delivery care led pregnant women to give births under the skilled birth attendants' supervision which contributed to lower the early infant mortality's risk. The overall available information assembled from our set of experiences indicated that methods of strategic importance consisted of the following: providing community health volunteers with reminders and others facilitating tools (i.e., pocket calendars for scheduling home visits, recording number and date of IFA tables' distribution and recollection at each visit; flyers to be used in interpersonal counselling etc.) thereby facilitating their role in supply and coverage; and recognizing the community health volunteers' role in programme progresses by the means of some kind of incentives such as allowances, certificate release, field‐testing of communication materials.

As far as health‐system workers were concerned, findings from our cases highlighted that establishing clear policy, providing rigorous training and regular‐refresher training, and disseminating dialogue/counselling guides, updated protocols and technical manuals concerning on IFA supplementation to health care personnel and providers were effective practices for the following: improving procurement and logistical management systems for maintaining supplements' availability at distribution points, thereby guaranteeing adequate supply and delivery of micronutrient supplements; and ensuring the appropriateness of health counselling messages thereby advocating supplements uptake.

##### Regular monitoring and surveillance of the systems

Timely collection and analysis of data for implementation monitoring and outcome evaluation (“performance evaluations” of provision, utilization and coverage, and “impact evaluations” of health outcomes and behaviours) were key activities highlighted within the set of case studies we reviewed. Establishing monitoring systems for all activities, through which accurate information flowed from the community up to the national level, resulted crucial to allow strategic solutions to be taken at the appropriate level in a timely manner. Efforts were put to support rigorous and continuous monitoring and communication on project progresses from both health‐workers and/or community agents to project‐technical responsible and from project‐team to commune/village/organization leaders. This allowed on one hand the constraints to effective implementation to be indentified and emended, on the other the community interest and commitment to be maintained. Furthermore, documenting impact and performance indicators on a regular basis, mostly by the means of external quality control systems, meant producing strong, and consistent evidence of on‐going effective implementation to confirm a gradual scale‐up. Evidence‐based approaches included surveys and analyses at programme baseline, midterm and the close concerning nutrition and implementation indicators of integrated package of interventions. Indeed, the adequacy evaluation of the MICAH programme (Berti et al., 2010) demonstrated that results‐based management relying on monitoring of numerous clinical, biochemical and behavioural indicators identified programme activities that were not producing positive outcomes, and allowed for midstream corrections, such as reducing geographic spread and intensifying the intervention in a smaller area to ensure all participants received all interventions. Interestingly, data were collected not only for results‐based management purposes but also as part of contractual requirements, thus had to be reported in detail to the donor guaranteeing the transparency of the process. The SUMMIT in Indonesia (Shankar et al., 2009) incorporated iterative, systematic enhancements into its intervention implementation, such as its revision of recruitment procedures for its Community Facilitator positions following analysis of interim data on inputs, activities, and outcomes.

## 5 CONCLUSIONS/RECOMMENDATIONS

Findings from our set of case studies showed that the positive impact on health reflected the successful achievement of IFA or MMN supplementation programmes in terms of coverage/supply, adherence and use, with task‐forces including multiple stakeholders and the existing health systems being the delivery‐platforms for reaching women. In particular, the implementation approaches which facilitated supplement delivery and adherence were context‐specific and focused mostly on the following: continuous educational and promotional activities; systematic and professional motivation and training of community health volunteers and health care workers; and dissemination of IEC materials and up‐to‐date technical guidelines delivering clear, standardized and specifically target messages. Piloting a project, that is carrying out operational research on several programme subcomponents and activities, helped for scaling the programme up, with all the strategies on design and management having been accompanied by careful monitoring and evaluation. In contrast, neglecting to document characteristics and impacts of implemented strategies in general and, mostly, when experiences failed, likely represented an obstacle towards the proper assessment of effectiveness and good practices, compromising the learning on how to adequately implement interventions. Taken together, the lessons learnt suggest that to successfully implement prenatal supplementation interventions and achieve sustainable solutions, major efforts must be put on developing delivery models appropriately to context, and providing regular documentation about outputs, outcomes and midcourse corrections of processes and tools, including clear description of practices promoted or approaches utilised.

## CONFLICTS OF INTEREST

The Authors declare no conflict of interest. C. Berti received financial support from WHO to prepare this paper.

## CONTRIBUTIONS

Research strategy: All authors contributed to prioritize the key‐topics to be reflected by the case‐studies and the strategy to be followed in order to identify relevant cases. CB collected available articles and reports; CB and MFG screened the retrieved documentation and identified eligible case‐studies. CB, MFG, ZAB, IC discussed and selected the most relevant case studies to be analysed.

Manuscript writing: CB analysed and interpreted data from the selected cases, wrote the first draft of the manuscript, attended the technical consultancy meeting, and finalized the manuscript accordingly; MFG contributed with data analysis and discussion, and with manuscript revision; ZAB and IC revised the manuscript. All authors approved the final version for publication.

## Supporting information

Appendix A: Program key‐characteristics gathered according to a simplified version of the *WHO/CDC Logic‐Model for Micronutrient Interventions in Public Health* (De‐Regil et al. 2014).
