# Supplementary material for: Multiple‐micronutrient supplementation: Evidence from large‐scale prenatal programmes on coverage, compliance and impact
Source: Matern Child Nutr. 2017 Dec 22;14(Suppl 5):e12531. doi: 10.1111/mcn.12531 (PMC6865895; doi:10.1111/mcn.12531)
Supplement: Supplementary file 1 — Appendix A: Program key‐characteristics gathered according to a simplified version of the WHO/CDC Logic‐Model for Micronutrient Interventions in Public Health (De‐Regil et al. 2014). [file MCN-14-e12531-s001.doc]

**Appendix A: Program key-characteristics** **gathered according to a simplified version of the *WHO/CDC Logic-Model for Micronutrient Interventions in Public Health* (De-Regil et al. 2014).**

This appendix summarises the detailed results on inputs, activities, outputs and outcomes concerning each selected case study.

***Nicaragua: The National Integrated Anemia Control Strategy (IACS) delivering iron-folic acid (IFA) supplementation to pregnant women through a multiple-comprehensive approach (Mora, 2007).***

| ***INPUTS and ACTIVITIES: Successful factors and implementation actions designed and applied*** |
| --- |
| - Adequate baseline country analysis on anaemia magnitude and "policies, environmental context and available resources" *status quo*; visibility of anaemia as a public health issue - Political and food industry commitment; partnerships with international and local cooperating, non-governmental agencies and organizations (USAID/MOST) for technical and financial support - Ownership by health-districts and local units; strong and effective health infrastructures and delivery services; community support and mobilization from community health volunteers - Solid BCC strategy; design and testing of persuasive communication materials - Intensive target-training, supervision and follow-up of personnel in different program components - Adequate management supply and delivery systems for program services - Incorporating IFA supplements into the official list of essential medicines; disseminating policies among health-care providers during training, and supplementation guidelines, protocols and communication materials for health-care workers; establishing effective procurement and logistical management systems for maintaining supplements' availability at distribution points, by standard procedures and training pharmacy staff - Establishment of a rigorous system for surveillance of the systems, including timely collection and analysis of performance and impact data |
| ***OUTPUTS: Coverage, adherence and implementation results*** |
| - Coverage: 70% in 2000; 85% in 2003-5 - Adherence in 2003-5: 58% for at least 6 months, 76% for ≥ 4 months - BCC in 2001: Awareness = > 90% women aware on anaemia importance-causes-consequences and how to take tablets (iron-absorption enhancers/inhibitors); 77% taught by health-care personnel on iron importance in pregnancy and childhood; 96% got clear instructions on how to take IFA tablets |
| ***OUTCOMES*** |
| Anaemia among women of reproductive age: 33.6% in 1993; 23.7% in 2000; 11.2% in 2003-5 |
| ***GAPS and/or CHALLENGES: Practical problems remained as potential constraints*** |
| - No data on anaemia prevalence among pregnant women even if they are IACS target; No data about trends in intermediate indicators (e.g. iron status); no updated data on dietary intake - Cost-effectiveness and sustainability not documented - Impact evaluation of BCC program not assessed over time - Lack of consensus/official policy on daily or intermittent IFAs policies for pregnant women among health-professionals → Use of non standard protocols - Insufficient resources for routine laboratory diagnosis → Poor compliance with policies on anaemia routine assessment - Lacking of IFA coverage data in pregnant women before 2000: No effective monitoring system for supply, distribution and local inventories → Reliable coverage at national, district and local level not regularly available |

BCC = Behavioural Change Communication

***Nepal: The National Iron Intensification Project (IIP) aiming to provide iron-folic acid (IFA) free of cost to pregnant women and breastfeeding mothers, as part of ante-natal and post-natal care services plus education on nutrition, health and hygiene (Pokharel et al. 2011).***

| **INPUTS and ACTIVITIES: Successful factors and implementation actions designed and applied** |
| --- |
| - Baseline-analysis of country anaemia situation; update on international anaemia-programming experiences; visibility of the nutrition problem - High political commitment; partnerships with international and local cooperating, non-governmental agencies and organizations for research, management, technical and financial support; stable funding from Department of Health budget and external donors (UNICEF, Micronutrient Initiative) - Strong and effective health infrastructures and delivery services; strong coordination in distribution; integrated community-based delivery of IFA by extensive network of motivated female community health volunteers - Program introduction supported by a local NGO through an orientation and awareness-raising event targeting District Health Office staff and district representatives of other ministries; carrying-out the initial training-to-trainers and supervising the cascade of skills-based training to health-workers and community health volunteers - Providing female community health volunteers with specifically designed tools such as registers to track their IFA supply and the numbers of pregnant women receiving IFA - Disseminating field-tested communication materials with simplified key messages about importance of anaemia and IFA benefits and availability among women, and posters, flipcharts and manuals among health-workers and community health volunteers in all program districts; promoting media-messages on anaemia during pregnancy on a regular basis; nationwide radio/TV campaigns on the IFA importance for pregnant women disseminated by the Government with UNICEF support - Coordinated management supply and logistic delivery systems for program services - Effective program supervision, monitoring and analysis system (i.e., verifying stocks, helping with stock planning, inquiring about any constrains to program performance); timely collection-analysis of ‘Performance’ and ‘Impact’ data - Carrying-out operational research as pilot studies supported by Micronutrient Initiative, and producing strong and consistent evidence of on-going effective implementation |
| ***OUTPUTS: Coverage, adherence and implementation results*** |
| - Coverage: Districts = 5 in 2003, 46 by 2007, 52 by 2008, 75 by 2010; Pregnant women = 23% in 2001, 59% in 2006, 81% in 2009, 80% in 2011 - Ante-natal care attendance: 49% in 2001, 74% in 2006, 87% in 2009 - Global attention as a successful scale-up of a national IFA supplementation-program for pregnant women |
| ***OUTCOMES*** |
| - Anaemia: 75% in 1998, 42% in 2006, 48% in 2011  - Median birth interval (months): 31.8 in 2001, 33.6 in 2006, 36.2 in 2009  - Maternal mortality ratio: 539 deaths per 100,000 live births in 1996 (95% CI = 392, 686), 281 in 2006 (95% CIs: 178, 384). |
| ***GAPS and/or CHALLENGES: Lack and practical problems remained as potential constraints*** |
| - No data about adherence - Inequalities - Lack of specific strategies to address maternal under-nutrition; lack of strategies focused on adolescent girls |

NGO = Non-Governmental Organization; IEC = Information, Education and Communication

***Vietnam: The National Iron-Deficiency Anaemia (IDA) Control Program including iron-folic acid (IFA) supplementation to pregnant women and non-pregnant women, as a part of a comprehensive approach (Ninh et al. 2003).***

| ***INPUTS and ACTIVITIES: Successful factors and implementation actions designed and applied*** |
| --- |
| - Political commitment; partnerships with universities and international agencies and organizations for overall assistance - Community health centers responsible for distributing supplements during ante-natal care visits or through collaborator visits; provision of supplements and IEC materials to schoolgirls by their teachers under healthcare-worker's supervision - [Primary health-care system delivering IFA tablets and ante-natal services for pregnant women growth monitoring for children](../../../../C:/Users/Cri/WHO%20paper/Ref/Ref%20to%20be%20shared/Vietn%20Phil%20Camb%20Thai%20II/Ninh%20Vietn%202003.doc) - Training and refreshing courses to health-workers - IEC as one of the highest priorities in the IDA program - Costs’ analysis |
| ***OUTPUTS: Coverage, adherence and implementation results*** |
| - Program Coverage: Pregnant women= 425000 in 1995, 46200 in 2000; Benefited districts = 46 in 1995, 17 in 2000  - Adherence and IEC: not documented |
| ***OUTCOMES*** |
| Anaemia: Pregnant women = 52.7% in 1995, 32.2% in 2000; Non-pregnant women = 40.2% in 1995, 24.3% in 2000 |
| ***GAPS and/or CHALLENGES: Practical problems remained as potential constraints*** |
| - Lack of supplements' supply and low community health volunteers utilization → Substantial decrease in program coverage - Difficulties in monitoring adherence; monitoring, supervision and reporting systems for supply, distribution and local inventories weak - Inequalities - Hookworm infection in pregnant women not documented* |

IEC = Information, Education and Communication.

* Nguyen et al., 2006

***Pilot studies from South-East Asia and Sub-Saharan Africa evaluating the impacts of iron-folic acid (IFA) supplements provided through delivery-platforms on process and/or health outcomes within women of reproductive age (both pregnant women and non-pregnant women).***

| **PHILIPPINES** (Nutrition Reviews, 2005) |
| --- |
| ***IMPLEMENTATION FEATURES: Operational Research*** |
| - Community-based social marketing framework and mobilization campaign with participation of government and industry. Supplements available at affordable prices from local drugstores, schools, local health-workers  - Strengthening municipal and village support for nutrition and health (i.e., local officials, health-personnel, village health-workers, teachers etc.). Use and improvement of service-delivery through existing health systems and pre/postnatal services (i.e., reminding pamphlet to take weekly tablets on Tuesday before bedtime eating foods rich in iron and vitamin C)  - Addressing supplementation adherence by a structured questionnaire *plus* count of empty pill-packets subjects turned. |
| ***OUTPUTS and OUTCOMES*** |
| Outputs: Adherence from the 1^st^ to the 4^th^ survey: Pregnant women = From 5.7 to 95.2; Non-Pregnant women = From 6.1 to 98.6  Outcome  - Women of reproductive age’s ferritin level higher with weekly *versus* daily supplementation  - Sales: Overall increase  - Awareness of IFA role in anaemia prevention for: Daily supplementation = High along the project; Weekly supplementation = Increase to over 80% by the end; Need to take IFA supplements throughout reproductive years = Increase to over 80% by the end  - Women knowledge and attitude: Iron and benefits of IFA supplementation = Marked increase by the 3^rd^ survey and positive attitudes of respondents asking for more information and feeling the need to take regularly IFA; Marked increase in knowledge on food-sources of iron |
| **VIETNAM** (Nutrition Reviews, 2005) |
| ***IMPLEMENTATION FEATURES: Operational Research*** |
| - Social mobilization and marketing strategies to introduce preventive IFA supplementation by creating attractive image for the supplements.  UNILAB supplements available at subsidized prices. Money from sales financed incentives for collaborators, management costs, regular communication activities within communes, revolving fund to purchase domestic IFA supplements at the project end  - Collaborative arrangement with Women’s Union network. Women’s Union collaborators trained on how to monitor sale and usage of tablets, how to collect and manage funds from sales, how to improve communication skills to motivate women through counselling and group discussions. High-school teachers trained to educate and communicate to women of reproductive age in school  - Campaigns informing target populations on project activities and promoting participation of actors involved in prevention of anaemia carried out with billboards, banners, promotional cars, loudspeakers used to communicate project’s messages. Monthly popular communication activities for improving KAP among women of reproductive-age at women’s clubs  - Continuous and impromptu communication and workshops between project-team and commune/village/organization leaders |
| ***OUTPUTS and OUTCOMES*** |
| Outputs: Small savings generated from selling the tablets, sufficient to expand the project only in two districts  Outcomes  - Anaemia: From 45.6 to 19.1% (p<0.0001); Iron deficiency: From 8.9 to 5.1%; IDA: From 8.6 to 1.0% (p<0.0001)  - KAP about nutritional anaemia and weekly IFA: Significant increase from the first months of the project  - Perception after regularly taking IFA tablets of: Feeling stronger, Being less tired, Sleeping better = Increase  - Sales = Rise from 54.5% to 92% in the first 6 months, followed by a sudden drop at the 9^th^ month (owing to rumours about IFA supplements as a potential cause of pregnancy interruption/diseases) which was reversed in the last 3 months |
| **INDONESIA** (Shankar et al. 2009) |
| ***IMPLEMENTATION FEATURES: Operational Research*** |
| - Scientific rigor of a randomized, double-blind, controlled clinical trial jointed to health-programs practicality  - Well-performing community facilitators: selection based on specific recruitment procedures enhanced *via* iterative examination of existing human resource practices, successes and failures of implementation and final outcomes; recruitment assessment to identify individuals with motivation, determination, integrity; training on use of field-manual, interview techniques, accurate data collection; SUMMIT-certification examinations to assess mastery of effective communications/enhanced social interactions, and technical knowledge; performance evaluated by a rating system; recruitment and certification procedures’ effectiveness assessed by examining the relationship between facilitator performance’s quality and supplementation’s impact on infant health within the facilitator’s cohort  - Direct information-exchange channel between SUMMIT-activities and pregnant women. Close collaboration between community-facilitators and health-service personnel comprising skilled birth attendants, pregnant women, informal/formal leaders to provide information to community on the value of pregnancy ante-natal care and delivery care. Beyond encouraging women to seek early ante-natal care and consume supplements, they assisted social marketing activities |
| ***OUTPUTS and OUTCOMES*** |
| Outputs^#^  - Adherence = 85.0%.  - Skilled birth attendants' use: From 35 to 53% in multiparous women; 63% among primiparous women  Outcomes  - Skilled birth attendants' use^#^ = 30% reduction in early infant mortality [RR (95% CI) = 0.70 (0.59- 0.83), p < 0.0001]  - Quality of community facilitators’ performance = better-performing improved the overall impact of MMN on early infant mortality [RR (95% CI) = 0.67 (0.49 - 0.92), p = 0.0117]; poorly performing derived no additional benefit from the MMNs |
| **VIETNAM** (Casey et al. 2010; Passerini et al. 2012) |
| ***IMPLEMENTATION FEATURES: Operational Research*** |
| - Distribution easily integrated into existing health-services; made freely available to all women  - Village health-workers: distribution of IFA supplements (and date recording) to individual women either through organized community meetings or home-delivery, counselling about IFA supplements collection on a monthly basis, possible side-effects and safe storage, delivery of educational materials*; Albendazole tablets administered either at the commune health station or supervised in the village by a commune health-worker  - From July 2006 to April 2009: Timely analysis of result about anaemia, iron status (Hb, Ferritin and sTfR assessed) and soil transmitted helminth infection; efficiency of distribution and women’s compliance evaluated by an independent Vietnamese NGO. Comparison of birth-weight between babies born in 2008 to women with access to the intervention and babies born in districts with no intervention implementation |
| ***OUTPUTS and OUTCOMES*** |
| - Anaemia: From 38 to 19%; Iron deficiency = From 19 to 6%; IDA = From 14 to 4%  - Low Birth Weight = 3% in intervention districts *versus* 7.4% in control districts (p = 0.017); Mean birth-weight = greater in the intervention districts (p < 0.001)  Outputs  - IFA table distribution from = From 99 to 81%  - Adherence = From 51 to 87% |
| **GHANA** (MacDonald et al., 2007) |
| ***IMPLEMENTATION FEATURES: Operational Research*** |
| MICronutrients And Health (MICAH) program. 1997 baseline survey.  - Strong partnership with Ministry of Health at district and sub-district levels  - IFA tablets ordered on a semi-annual basis by MICAH. IFA distribution by sub-district Health-Teams providing maternal and child services to communities; supported by a network of community health volunteers established by MICAH and supervised by Ministry of Health staff  - Regular training and capacity building opportunities for community health volunteers on counselling women about IFA; small incentives in recognition of their role in the program-success  - Strong nutrition education component: messages on anaemia and importance of taking regular IFA supplements through radio talks and presentations in mosques, churches, and marketplaces; clear information to improve adherence (i.e., possible side-effects, ways to manage them, remembering tips to take tablets according to dosing schedules)  - Cross-sectional surveys at mid-term and close of the program, including comparison groups from non-MICAH communities (control districts): Anaemia, Hb, Malaria; weekly IFA coverage or IFA coverage during current pregnancy |
| ***OUTPUTS and OUTCOMES*** |
| Outputs: Coverage (%): Pregnant women = 21.7 in 1997, 69.7 in 2000, 69.2 in 2004 [65.2 in control districts; p < 0.05]; Women of reproductive age = n.a. in1997, 93.9 in 2000, 75.7 in 2004 [1.6 in control districts; p < 0.05]  Outcomes: Anaemia (%): Pregnant women = 60.0 in 1997, 48.4 in 2000, 18.2 in 2004 [35.9 in control districts; p < 0.05]; Women of reproductive age = 47.9 in 1997, 26.9 in 2000, 16.1 in 2004 [32.3 in control districts; p < 0.05] |

^*^ Phuc et al., 2009; ^#^ Independently of the supplement used.

IEC = Information, Education and Communication; KAP = Knowledge, Attitudes and Practices; UNILAB = United Laboratories (in the Philippines); IDA = Iron-Deficiency Anaemia; NGO(s) = Non-Governmental Organization(s); RR = Relative Risk; Hb = Haemoglobin; sTfR = soluble Transferrin Receptor
